# Cwc21p promotes the second step conformation of the spliceosome and modulates 3′ splice site selection

**DOI:** 10.1093/nar/gkv159

**Published:** 2015-03-03

**Authors:** Amit Gautam, Richard J. Grainger, J. Vilardell, J. David Barrass, Jean D. Beggs

**Affiliations:** 1 Wellcome Trust Centre for Cell Biology, University of Edinburgh, King's Buildings, Mayfield Road, Edinburgh, EH9 3BF, UK; 2 Department of Molecular Genomics, Institute of Molecular Biology of Barcelona (IBMB), 08028 Barcelona, Spain; 3 Institució Catalana de Recerca i Estudis Avançats (ICREA), 08010 Barcelona, Spain

## Abstract

Pre-mRNA splicing involves two transesterification steps catalyzed by the spliceosome. How RNA substrates are positioned in each step and the molecular rearrangements involved, remain obscure. Here, we show that mutations in *PRP16, PRP8, SNU114* and the U5 snRNA that affect this process interact genetically with *CWC21*, that encodes the yeast orthologue of the human SR protein, SRm300/SRRM2. Our microarray analysis shows changes in 3′ splice site selection at elevated temperature in a subset of introns in *cwc21Δ* cells. Considering all the available data, we propose a role for Cwc21p positioning the 3′ splice site at the transition to the second step conformation of the spliceosome, mediated through its interactions with the U5 snRNP. This suggests a mechanism whereby SRm300/SRRM2, might influence splice site selection in human cells.

## INTRODUCTION

Pre-mRNA splicing is essential for gene expression in eukaryotes and is catalyzed by the spliceosome. During spliceosome assembly, five small nuclear RNA–protein complexes (snRNPs) U1, U2, U4, U5 and U6 assemble in an orderly manner with the pre-mRNA (1–3). In addition, many non-snRNP proteins associate with the spliceosome at different stages of the process (3). The U1 snRNP identifies and binds to the intronic sequence next to the 5′ splice site (5′SS) and the U2 snRNP associates at the branch site to create a precursor spliceosome complex A. Even before complex A formation the choice of splice sites has generally been made, with the 3′ splice site (3′SS) often being at the next AG dinucleotide downstream of the branch site(4). However, there is evidence that, in some cases, 3′ splice site choice can occur after the first step of catalysis (5,6). The U4, U5 and U6 snRNPs form a tri-snRNP complex prior to associating with the forming spliceosome. A conformational change then displaces the U1 and U4 snRNPs and the U2 and U6 snRNAs associate to form part of the catalytic centre (B^act^ spliceosomal complex) (7) while the NineTeen Complex (NTC) of proteins stabilizes interaction of U5 and U6 snRNPs with the assembled spliceosome. The B^act^ complex is then catalytically activated by Prp2p and Spp2p to form B* complex (8), in which the branch site adenosine is exposed, ready for catalysis.

Pre-mRNA splicing involves two steps; cleavage at the 5′SS produces free 5′exon and intron-3′exon in lariat form, then the 3′SS is cleaved and the two exons are joined. Between the two reaction steps, the substrate has to be repositioned in the catalytic centre. This involves a number of RNA rearrangements that require at least six factors: Prp8p, Prp17p, Prp18p, Slu7p and two DEAH-box ATPases, Prp16p and Prp22p (9,10). Konarska *et al*. (11,12) proposed a model, invoking a competition between the first and second step conformational states of the catalytic centre, such that the first state conformation needs to be destabilized in order to favour the second step of splicing and *vice versa*. In this model, mutations that destabilize one conformation of the spliceosome favour the other, altering the balance of the splicing process and affecting fidelity. Prp16p is thought to destabilize U2/U6 helix I after step 1, acting as a fidelity factor by promoting rearrangements that allow the correct 3′SS to enter the catalytic centre for the second step (13). The cold-sensitive *prp16-302* mutant inhibits the second step of splicing and can be rescued by destabilizing U2/U6 helix 1 or by depleting Isy1p (14). At step 2, Prp22p functions as a fidelity factor and promotes the release of the spliced exons from the spliceosome (15,16).

Photochemical cross-linking of the U5 snRNP protein, Prp8p, to the 5′SS, the branch site and the 3′SS in the pre-mRNA lead to the proposal that Prp8p acts as a cofactor at the site of RNA catalysis ((9) and references therein). Moreover, structural studies showed that this large protein forms a cavity within which the RNA-mediated splicing reactions take place (17,18). Prp8p binds to the stem-loop 1 region of U5 snRNA. U5 loop 1 is evolutionarily invariant and interacts with exon sequences adjacent to the 5′ splice site before the first step of splicing (19) and with both exons for their correct alignment and joining in the second step (6,20–23). These RNA interactions do not involve Watson–Crick base pairing and are thought to be stabilized by proteins, including Prp8p (9,24–26).

Prp8p also interacts both physically and genetically with the B^act^ protein, Cwc21p (27). Cwc21p was shown to be a yeast orthologue of human splicing factor SRm300/SRRM2, a component of the spliceosome's catalytic core (2,27–28). Cwc21p is a small 135-residue protein with a conserved cwf21 domain through which it binds to Prp8p. Isy1p, recruited to the spliceosome as a part of the NTC prior to Cwc21p (8,29), genetically interacts with it. Isy1p is required for the splicing of certain suboptimal introns (30) and was proposed to function as a fidelity factor (14). Although neither *CWC21* nor *ISY1* is essential for viability, *cwc21Δ* is lethal in combination with *isy1Δ* at 37°C, suggesting that Cwc21p and Isy1p have related functions (27,31). However, by itself *isy1Δ* has a mild first step splicing defect, accumulating *ACT1* pre-mRNA at 37°C, whereas *cwc21Δ* has a mild second step splicing defect (27).

Little is known about how interactions in the catalytic centre are stabilized or how they can be modified to alter splicing fidelity or splice site usage. Here we show that *CWC21* displays genetic interactions with components of the catalytic centre of the spliceosome, supporting a role for Cwc21p in promoting step 2 catalysis. Our data indicate that Cwc21p affects 3′SS selection, probably by influencing exon alignment in the catalytic centre. This suggests a mechanism whereby SRm300/SRRM2 might influence splice site selection in human cells.

## MATERIALS AND METHODS

*Saccharomyces cerevisiae* strains and plasmids are listed in Supplementary Table S1. Yeast manipulations were performed using standard laboratory procedures. Deletion of *CWC21* on the yeast genome was done by one step transformation (32) and confirmed by polymerase chain reaction (PCR). Mutagenesis of plasmids was achieved using the quickchange protocol (Stratagene) and checked by sequencing.

### Plasmid shuffle

All plasmid shuffle experiments involved selecting three colonies transformed with the mutant allele. Following growth in selective liquid media to stationary phase, 0.3 ODs of each colony were spotted on 5-fluoroorotic acid (5-FOA) (0.1% w/v) plates and grown at 30°C. Strains that grew on 5-FOA were grown in selective medium and 0.3 ODs of each colony were spotted on Yeast extract Peptone Dextrose Adenine (YPDA) plates followed by growth at the specified temperatures.

### Yeast two-hybrid assays

Yeast two-hybrid assays were performed using the haploid L40ΔG yeast strain co-transformed with pACT2-Cwc21 bait and LexA-Snu114 prey plasmids, selecting for expression of the *HIS3* reporter gene (growth on medium lacking leucine and tryptophan to select for both plasmids and lacking histidine to select for *HIS3* expression; -LWH medium) (33) and including various concentrations of 3-aminotriazole (3-AT) that increases the stringency of the *HIS3* activation, allowing an assessment of the strength of interaction.

### Copper reporter assays

Copper growth assays were performed as described (34). *ACT1-CUP1* (*LEU2*) reporter plasmid was transformed into *cup1Δ* strain yJU75-8+ with or without *cwc21Δ* and selected on -leu plates containing various concentration of CuSO_4_.

### Microarray analysis and quantitative PCR

The design of the microarray was essentially as described previously (23) but, in addition to annotated splice junctions, contained oligo probes for known alternative 5′ or 3′ splice sites (35,36) and predicted alternative 3′ splice sites (37), as well as a probe for the most 3′ exon of each intron-containing transcript. The arrays were printed at the Division of Pathway Medicine, University of Edinburgh. WT and *cwc21Δ* cells were grown at 30°C and shifted to 37°C for 60 min prior to extraction of RNA (38). Roche transcriptor was used according to the manufacturer's instructions to produce cDNA. The real-time PCR was performed with Invitrogen Express SybrGreen as per the manufacturer's instructions on a Stratagene Mx3005P. The analysis was performed in biological duplicate, with dye reversal. The splice junction signals were standardized to the 3′ exon signal of the same transcript (to compensate for the RNA level) and then as a ratio of mutant to wild-type (WT). The list of probes and the microarray results are available in the ArrayExpress database (www.ebi.ac.uk/arrayexpress) under accession number E-MTAB-3240.

## RESULTS

### Genetic interactions of *CWC21* and *PRP16* suggest a role in promoting step 2

**Figure 1.**
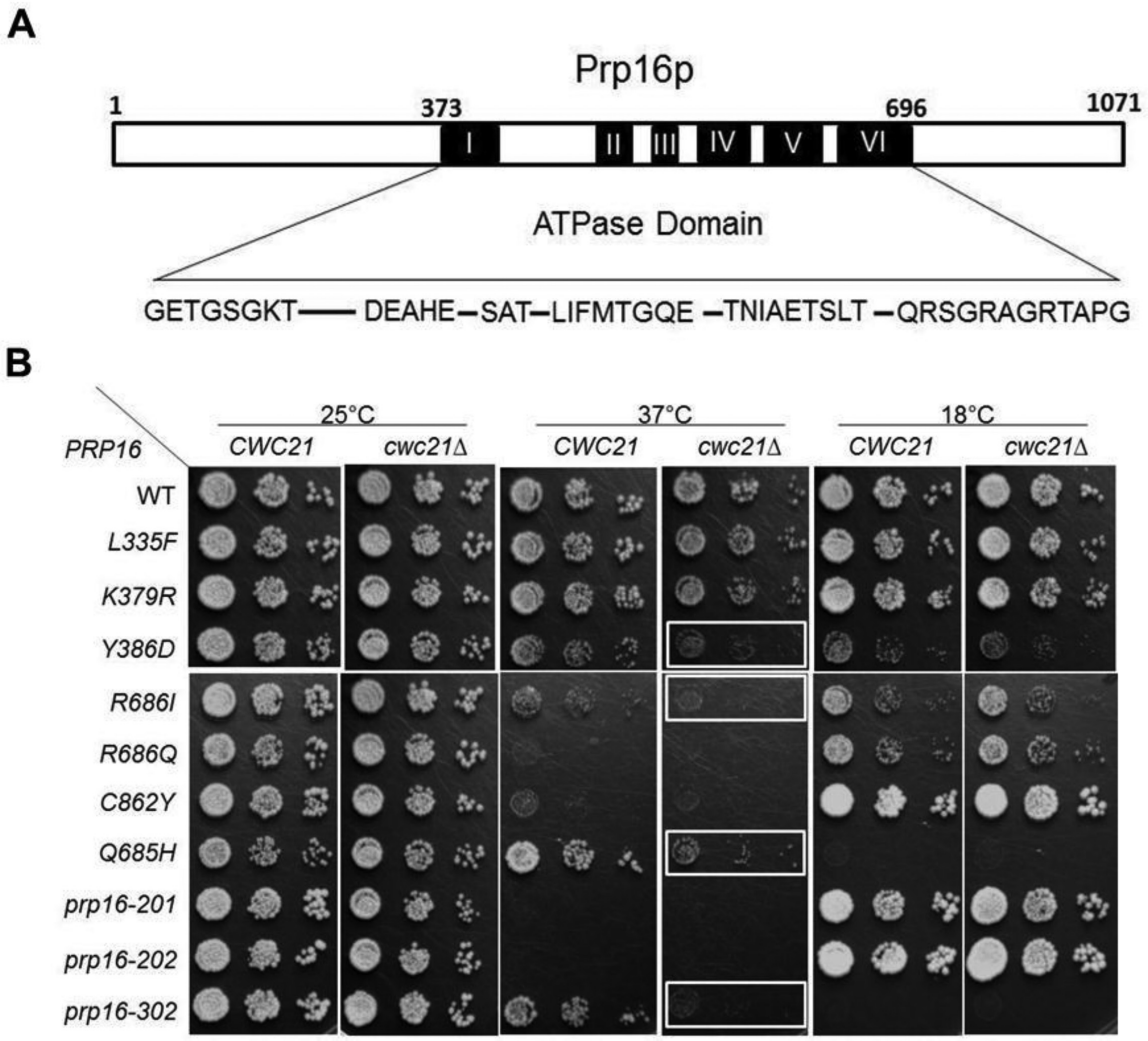
Genetic interaction of *cwc21Δ* and *PRP16* mutants. (**A**) Schematic representation of Prp16p highlighting the ATPase domain, with various alleles that were tested in the presence or absence of *CWC21*. Numbers refer to amino acid residues in Prp16p from *Saccharomyces cerevisiae*. Amino acids in each motif are shown. (**B**) *CWC21* has genetic interaction with *PRP16*. Plasmids containing the genomic *PRP16* sequences with mutations as indicated were transformed into PRP16KO or PRP16KO-21*Δ* (*cwc21Δ*) plasmid shuffle strains. Cultures were adjusted to *A*_600_ of 0.3 and 10-fold serial dilutions were spotted onto YPDA agar plates and incubated at 25 and 37°C for two days and 18°C for 5 days. The boxes indicate observed growth defects.

*ISY1*, which interacts genetically with *CWC21*, also interacts genetically with *PRP16* (14). We therefore investigated the effect of combining *cwc21Δ* with various alleles of *PRP16* that have mutations in the ATPase domain (Figure 1A). *cwc21Δ* showed heat sensitivity in combination with *prp16-1* (Y386D), *prp16-Q685H, prp16-R686I* and especially, with *prp16-302* (R456K-G691R) (Figure 1B, boxed). The *prp16-1* and *prp16-302* alleles cause a second step defect (14,39). The Q685H and R686I defects are less well characterized but *prp16-R686I* is lethal in combination with *prp8-R1753K* that inhibits step 2 (40). These results are compatible with *cwc21Δ* also inhibiting step 2 and, conversely, supports a role for Cwc21p in promoting step 2. This suggests that Isy1p and Cwc21p promote different conformational changes in the spliceosome and, consistent with this idea, *cwc21Δ* did not rescue the cold-sensitive growth defect of *prp16-302* (Figure 1) that is suppressed by *isy1Δ* (14).

### *CWC21* interacts genetically with U5 snRNA loop I and *PRP8*

**Figure 2.**
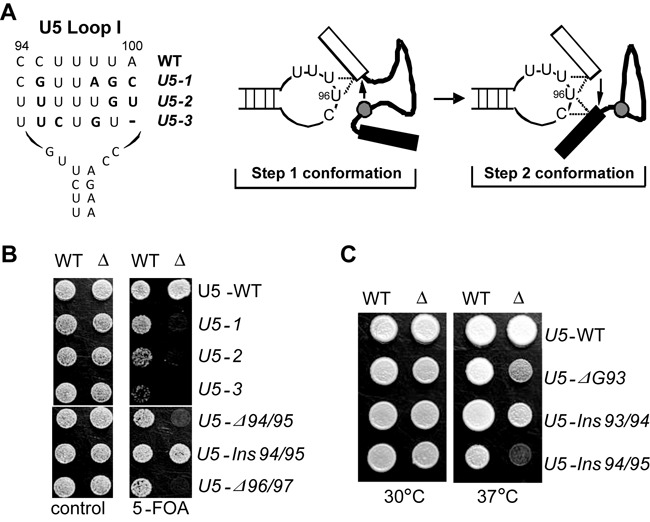
Genetic interactions of *cwc21Δ* with U5 snRNA loop I alleles. (**A**) Diagram of yeast U5 snRNA loop I. Three U5 alleles are aligned below the WT sequence with alternative nucleotides highlighted in bold. The U5 loop I interaction with the exons before and after step I of splicing (according to (41)) are shown to the right and dashed lines represent known crosslinks. (**B**) Mutations in U5 snRNA loop I show synthetic lethality in the absence of *CWC21*. (**C**) Mutations in U5 snRNA loop I show temperature-sensitive defects in the absence of *CWC21*.

Loop 1 of the U5 snRNA affects the alignment of exons in the spliceosome (21,41) (Figure 2A). We found that three U5 loop I mutations, *U5-1, -2, -3* (Figure 2A), that affect the splicing of distinct subsets of pre-mRNAs and cause slow growth at 37°C (23,42) are synthetic lethal with deletion of *CWC21* (Figure 2B). Also, the U5 loop I deletion mutants *Δ94/95* (deletion of CC at positions 94 and 95) and *Δ96/97* (deletion of UU at positions 96 and 97) that reduce the efficiency of step 2 by ∼50% (21), were lethal in conjunction with *cwc21Δ*, suggesting a role for Cwc21p in promoting step 2. The *U5-ΔG93* and *U5-Ins^1U^94/95* (insertion of an additional U between positions 94 and 95) alleles, although not lethal in combination with *cwc21Δ*, caused sensitivity to raised temperature, whereas there was no apparent effect with *U5-Ins^1U^93/94* that is nearer one end of the loop (Figure 2C). These allele-specific effects are compatible with a role for Cwc21p helping to align or stabilize the exon substrates in the catalytic centre of the spliceosome.

**Figure 3.**
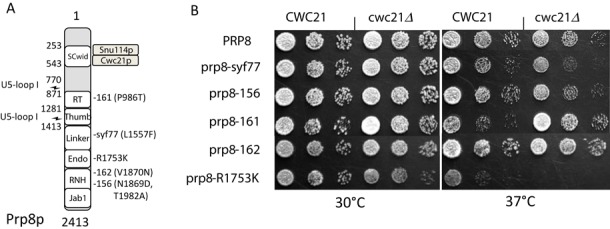
Genetic interaction of *cwc21Δ* and *PRP8* mutants. (**A**) Schematic representation of Prp8p showing the various positions of the *prp8* alleles tested in the presence or absence of *CWC21*; numbers refer to amino acid residues from *Saccharomyces cerevisiae*. The direct binding sites of Cwc21p and Snu114p to Prp8p's SCwid domain are shown (9,27) along with sites of UV-crosslinking to U5 loop 1 (44). The Reverse Transcriptase-like (RT), Thumb, Linker, Endonuclease (Endo), RNase H (RNH) and Jab1 domains are indicated (17). (**B**) *cwc21Δ* interacts genetically with *prp8* alleles. Cultures with or without *CWC21* were adjusted to *A*_600_ of 0.3 and 10-fold serial dilutions were spotted onto YPDA agar plates and incubated at the indicated temperatures.

Prp8p binds to loop 1 of U5 snRNA and appears to stabilize the interaction of loop 1 with the exons (43,44). We therefore tested the effect of deleting *CWC21* in combination with different *prp8* alleles (Figure 3A). Of three alleles, *prp8-156, prp8-161* and *prp8-162*, that inhibit the first step of splicing, *prp8-161* (P986T) showed improved growth at 37°C when combined with *cwc21Δ* compared with either mutation alone (Figure 3B) and the more C-terminal *prp8-156* and -*162* showed little, if any effect. In contrast, *prp8-R1753K* and *prp8-syf77* (L1557F) that inhibit the second step of splicing (40), displayed an exacerbated heat sensitivity in combination with *cwc21Δ* (Figure 3B). These effects are compatible with Cwc21p favouring the step 2 spliceosomal conformation. It has been proposed that the *prp8-R1753K* mutation loosens interactions between U5 loop I and the exons (45). Therefore, the enhanced growth defect that results from combining *cwc21Δ* with *prp8-R1753K* further supports a role for Cwc21p in stabilizing the exons in the catalytic centre.

### *CWC21* deletion suppresses the *snu114-40* growth and splicing defect

**Figure 4.**
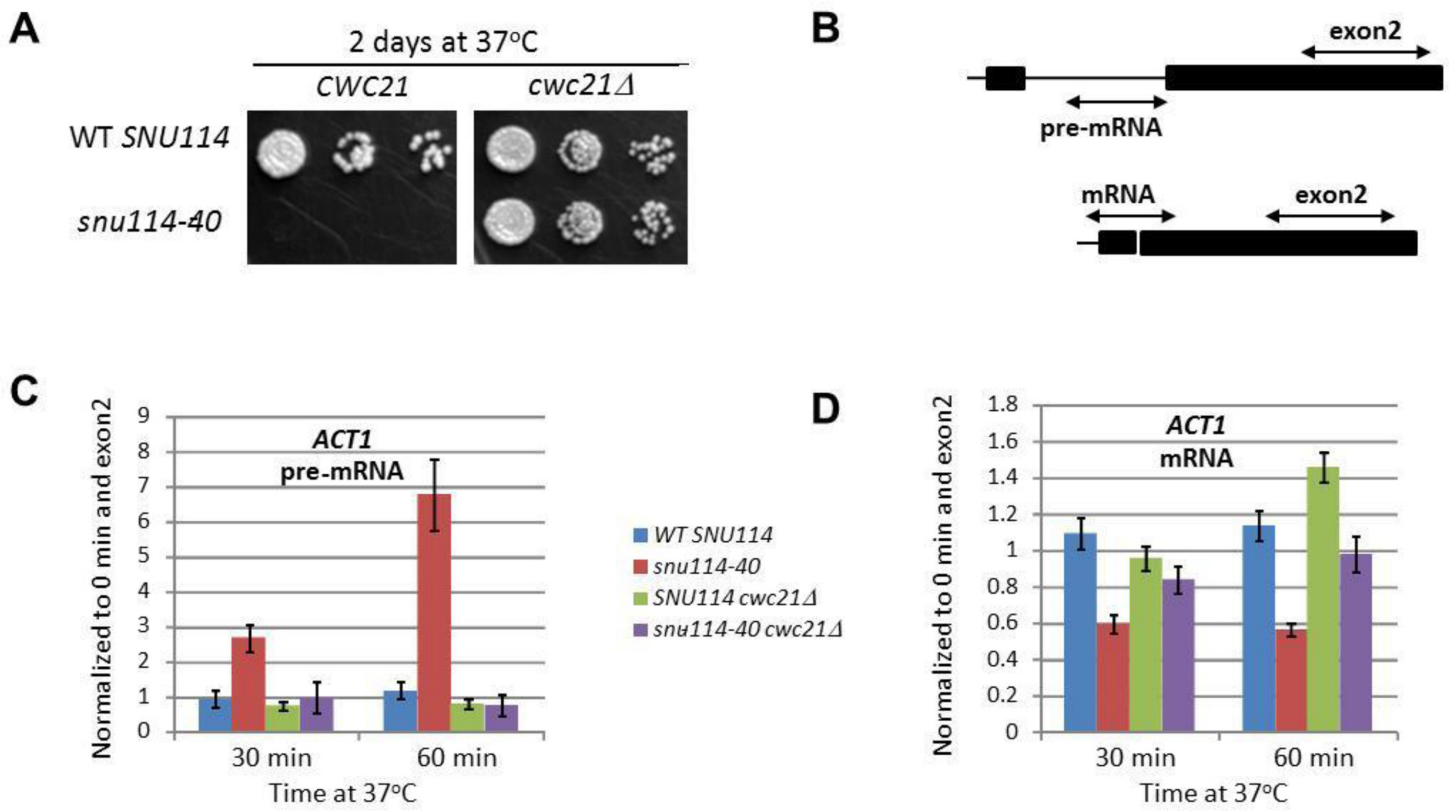
*CWC21* deletion suppresses the *snu114-40* splicing defect. (**A**) Deletion of *CWC21* rescues heat sensitivity of *snu114-40*. After shuffling out the WT plasmid yeast cells were grown to stationary phase in YPDA medium. The cells were grown to log phase at 30°C and shifted to 37°C. Then, cells with OD_600_ of 0.3, 0.03 and 0.003 were spotted on YPDA plates and grown at 30 or 37°C. (**B**) Diagram showing different sets of primers used for reverse transcriptase-qPCR (RT-qPCR) analysis of *ACT1* RNA (**C**). RT-qPCR analysis of *ACT1* RNA extracted from WT *SNU114* and *snu114-40* cells shifted from 30 to 37°C for the indicated times in the presence or absence of *CWC21*. Pre-mRNA and mRNA levels are plotted after normalizing to time point zero and respective exon 2 levels.

A role for Cwc21p in stabilizing the exons in the spliceosome could explain the mild second step splicing defect with *cwc21Δ* at elevated temperature (27). Therefore, we hypothesized that if Cwc21p has a significant role in stabilizing the step 2 conformation, *cwc21Δ* spliceosomes should be biased towards the step I conformation. The *snu114-40* (M842R) mutation blocks splicing before step I (46) and causes heat-sensitive growth. Significantly, we found that deletion of *CWC21* rescues the *snu114-40* growth defect (Figure 4A), indicating that the lack of Cwc21p favours the step I conformation. Interestingly, residue M842 lies in domain IVa of Snu114 and this domain is sufficient for Cwc21p binding in a yeast two-hybrid assay (Supplementary Figure S1 and (27)).

To test if this rescue in growth is due to suppression of the splicing defect of *snu114-40*, we monitored splicing in *snu114-40* cells in the presence or absence of *CWC21*. In RT-qPCR analysis (Figure 4B), RNA extracted from *snu114-40* cells that had been incubated at the restrictive 37°C for 30 or 60 min showed strong accumulation of *ACT1* pre-mRNA compared to RNA from cells grown at the permissive temperature (0 min) and compared to RNA from WT cells (Figure 4C). In addition, the level of mRNA in the *snu114-40* mutant decreased to 50% that of WT (Figure 4D), as expected for a splicing defect before the first catalytic step. In contrast, the *snu114-40, cwc21Δ* double mutant showed no pre-mRNA accumulation at 37°C and the level of *ACT1* mRNA was similar to WT (Figure 4C and D) consistent with the notion that the step I splicing defect caused by *snu114-40* is suppressed by the absence of Cwc21p in the double mutant strain. Furthermore, this supports our previous proposal that Snu114p and Cwc21p function together in the catalytic centre of the spliceosome (27).

### *cwc21Δ* affects splicing accuracy

**Figure 5.**
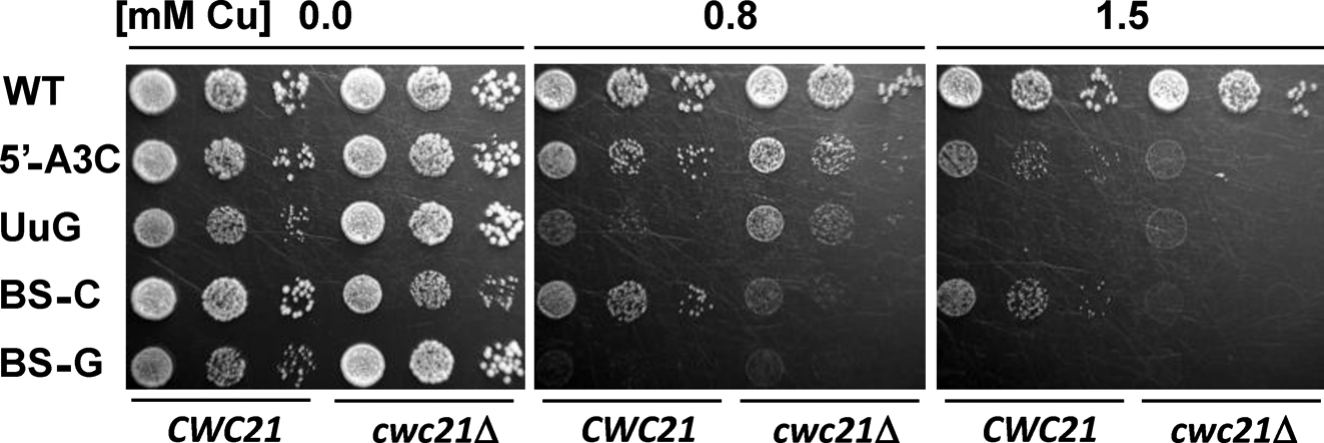
*cwc21Δ* affects splicing accuracy. Yeast strains carrying different *ACT1-CUP1* reporters containing mutated sequences at catalytically important residues were spotted in a series of 10-fold dilutions on media containing different concentrations of CuSO_4_. Their copper tolerance is an indication of the efficiency of splicing the reporter transcripts in the presence or absence of *CWC21*.

Isy1p was proposed to have a role in the regulation of splicing fidelity during the Prp16p-dependent transition toward the second step of splicing (14). To investigate whether *cwc21Δ* affects the splicing of mutant introns that are defective in step 1 or step 2, *ACT1-CUP1* reporter constructs were tested that contain mutations in the 5′SS, branch site (BS) or the 3′SS region of the *ACT1* intron. Efficient splicing of the *ACT1-CUP1* transcript is required for growth on medium containing copper sulphate. The 5′SS-A3C mutation hyperstabilizes the 5′SS/U6 duplex by interacting with U6-G50, resulting in an inefficient transition between the first and second conformations of the catalytic centre and inhibiting the second step of splicing (11). The 5′SS-A3C defect was enhanced by *cwc21Δ* (Figure 5), compatible with *cwc21Δ* further inhibiting the second step. The BS-C mutation is limiting for both steps of splicing (40). Its defect is exacerbated by mutations, including *isy1Δ*, that disrupt U2/U6 helix I (13) and is enhanced by *cwc21Δ* (Figure 5). The BS-G and UuG mutations destabilize the second step conformation of the spliceosome, causing accumulation of first step products (11,40). These highly expressed, second step mutant reporters are detrimental to growth of WT cells even in the absence of copper but are better tolerated with *cwc21Δ* (Figure 5). Also, growth in the presence of copper was slightly and reproducibly, improved by *cwc21Δ*. This is consistent with *cwc21Δ* relaxing the second step requirements, thereby suppressing the toxic effects of overexpressing BS-G or UuG transcripts. Thus, *cwc21Δ* relaxes the stringency of BS-G and of UuG 3′SS use even though these mutations reduce the second step of splicing.

### *cwc21Δ* promotes unusual splicing events under thermal stress

**Figure 6.**
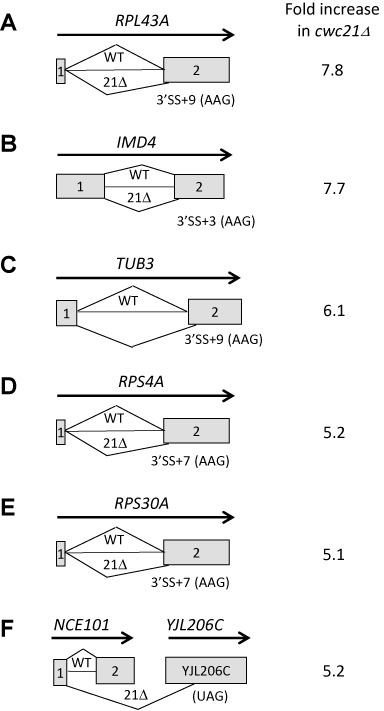
*cwc21Δ* promotes unusual splicing events under thermal stress. (**A**–**F**) Schematic representations of alternative splicing events detected by microarray analysis to occur at least five-fold more frequently in *cwc21Δ* (*21Δ*) than in WT. Numbered boxes represent exons and thin lines represent introns and splicing events. Positions of alternative 3′SS are indicated with the number of bases relative to the annotated 3′SS, with the last three bases in parentheses. Numbers on the right indicate the fold increase in *cwc21Δ* relative to WT from microarray data that are presented (in biological duplicate) in Supplementary Table S2.

To investigate the effect of *cwc21Δ* on splice site use more generally, microarray analysis was performed. In addition to probes that detect mRNAs with canonical splice junctions (as annotated in the Saccharomyces Genome Database; http://www.yeastgenome.org/), the microarray contained probes to detect splice junctions that would be formed using known alternative 5′ or 3′ splice sites (35,36) or predicted alternative 3′ splice sites (37). RNA was analysed from WT and *cwc21Δ* cells after incubation at 37°C for 1 h in order to provide conditions of mild thermal stress. The splicing of most transcripts was unaffected by the *cwc21Δ* mutation, however, six mRNA species were detected at a greater than five-fold increase in *cwc21Δ* cells compared to WT (Figure 6; for the full dataset see Supplementary Table S2) and were spliced using cryptic 3′ splice sites. Five of these splicing events involved use of an AAG 3′SS a few bases downstream of the canonical 3′SS (Figure 6A–E). The sixth alternative splicing event was a product of the first exon of *NCE101* being spliced to a downstream open reading frame (ORF), *YJL206C* (Figure 6F). Sequencing showed that this was identical to a cDNA reported by Miura *et al*. (36) that encodes a hybrid ORF containing parts of *NCE101* and *YJL206C*. RT-PCR detected the *NCE101–YJL206C* hybrid transcript at a low level in WT cells at 37°C but much more abundantly in *cwc21Δ* cells after shifting from 30 to 37°C for 30 min (Supplementary Figure S2A).

Sequencing of *TUB3* cDNAs identified an additional unexpected splicing event (for which there was no probe in the microarray) using a CUG 3′SS within the *TUB3* intron, 26 nt downstream from the BP and 113 nt upstream of the annotated 3′SS (Supplementary Figure S2B). Fewer cDNAs with this structure were obtained from RNA from WT cells than from *cwc21Δ* cells at 37°C, suggesting that the unusual splicing event may be more frequent in the mutant cells. Aberrant splicing of *TUB3* transcripts likely explains the increased benomyl sensitivity of the *cwc21Δ* strain at 37°C (Supplementary Figure S2B).

## DISCUSSION

It has been demonstrated that for optimal splicing, introns with BP to 3′SS distances greater than about 45 nt require secondary structure to reduce the effective distance between these functional sites in the RNA (47,48). In these situations, RNA helicases that modify RNA secondary structures may play an important role (49). In the case of *TUB3* transcripts, the unusually long distance (139 nt) from the BP to the annotated 3′SS may make 3′SS selection more susceptible to errors, especially at elevated temperature, which can make alternative splice sites more accessible (37,50). Four of the six alternatively spliced transcripts that were enriched with *cwc21Δ* have long BP to 3′SS distances. Two (*RPL43A* and *RPS30A*) do not, but these have suboptimal 5′SS sequences (GUAUGA and GUACGU respectively; underlined base is suboptimal). It has also been proposed that the exon sequences that flank the splice sites influence splice usage (51). By this criterion, the UUG sequence that follows the annotated 3′SS of *TUB3* is poor in supporting 3′SS selection, however, this consideration appears to apply only to *TUB3*. We propose that Cwc21p alters the ability of the U5 snRNP to align the exons in the catalytic centre of the spliceosome, which is affected by a number of different criteria depending on the transcript, In this scenario, although Cwc21p is not normally essential for splicing, its function becomes more important for the correct splicing of a subset of introns at elevated temperature, due to its effect in stabilizing molecular interactions in the catalytic centre.

### A role for Cwc21p in substrate positioning

Between the first and second catalytic reactions, destabilization of the highly conserved U2/U6 helix 1 in the spliceosome was postulated to lead to the formation of a non-catalytic ‘open’ state in which a ‘substrate re-positioning’ step allows the 3′SS to interact with U5 loop 1, followed by reformation of U2/U6 helix 1 that ‘closes’ the catalytic centre to promote step 2 (13). At the same time, Prp16 activity permits binding of three step 2 factors, Slu7p, Prp18p and Prp22p that may promote other conformational re-arrangements (52). Data presented here, together with published results showing poor growth when *cwc21Δ* is combined with *slu7-ts1, prp22-1* or *prp18-ts* (53), support a role for Cwc21p in forming or stabilizing the step 2 catalytic centre.

The strong synthetic lethality observed when *cwc21Δ* was combined with U5 loop 1 mutations suggests a major impact of Cwc21p on U5/exon alignment. In particular, it can explain the increased usage of inappropriate 3′ splice sites that we observed not only with mutant reporter transcripts but also with endogenous transcripts. We propose that Cwc21p affects substrate re-positioning during the transition from step 1 to step 2. In principle, Cwc21p could similarly affect substrate positioning prior to step 1. In our microarray screen for the use of non-canonical splice sites, although no examples of alternative 5′SS were observed, the range of alternative 5′SS probed was far from exhaustive and so the use of alternative 5′SS is not ruled out. A role for Cwc21p in modifying exon alignment suggests a mechanism whereby Cwc21p could promote the splicing of certain introns, especially where intron features are suboptimal for splicing.

Prp8p forms a cavity that surrounds the catalytic centre of the spliceosome (17). Prp8p contacts the ends of the exons near the splice sites, stabilizing their interaction with U5 loop1 and many mutations in Prp8p affect splice site use (reviewed in (9)). The size of U5 loop 1 is critical for correct juxtaposition of the splice sites (21) but the mechanism of exon alignment is unclear. The loop 1 sequence is very highly conserved and appears to be important for binding Prp8p (23,42–43). As shown here, deletion of *CWC21* is lethal in combination with U5 loop 1 mutations. Although RNA binding by Cwc21p has not been ruled out, its sequence does not contain a recognizable RNA binding motif. A likely scenario is that Cwc21p exerts its effects through its direct interactions with Prp8p and Snu114p (27). We propose that Cwc21p modulates the interactions of Prp8p and Snu114p (and potentially of other factors (6)) with the substrate RNA in the catalytic centre, thereby affecting exon alignment during substrate positioning (before 5′ splice site cleavage) and/or repositioning (before exon ligation). *CWC21* is non-essential under normal growth conditions, however, lack of Cwc21p causes splicing defects at elevated temperature. Presumably, the absence of Cwc21p under these conditions results in the misalignment of exon/U5 loop I, resulting in errors. By affecting the stability of molecular interactions in the catalytic centre, Cwc21p may also influence the relative stability of the different conformational states. Indeed, this might explain the suppression of the *snu114-40* first step defect by *cwc21Δ*, however, this suppression is specific for the *snu114-40* allele (data not shown), which lies in the region of Snu114p that interacts with Cwc21p. Therefore, Cwc21p may directly modify Snu114p function.

### Cwc21p and Isy1p

Certain properties of *CWC21* and *ISY1* suggest that they may act at the same step(s): (i) *cwc21Δ* enhances the step 1 splicing defect of *isy1Δ* (27,31); (ii) these deletion mutations affect expression of *ACT1-CUP1* reporters similarly, reducing use of the BS-C mutant reporter and enhancing use of the UuG 3′SS reporter. However, they behave differently in that: (i) by itself *isy1Δ* has a mild first step splicing defect with *ACT1* pre-mRNA at 37°C whereas *cwc21Δ* has a mild second step splicing defect (27); (ii) *isy1Δ* suppresses the cold-sensitive growth defect caused by the *prp16-302* allele whereas *cwc21Δ* does not. Isy1p, although not essential, is required for the splicing of certain suboptimal introns (30) and for efficient splicing of *ACT1* transcripts both *in vitro* (14) and *in vivo* (27). It was proposed to function as a fidelity factor and several possible modes of action have been suggested, including stabilization of the step 1 conformation of the spliceosome, negative regulation of Prp16 ATPase activity or more directly, in surveillance of 3′SS selection at the exon ligation step (14). Overall, the available data suggest that Isy1p promotes the first step of splicing, whereas Cwc21p primarily promotes the second step but may affect both steps. Isy1p is thought to function by slowing the transition from the first to the second step of spicing (14). Therefore, Isy1p and Cwc21p may have distinct functions during the transition state between step 1 and step 2, potentially acting in opposition, although we do not exclude an additional function where Cwc21p and Isy1p may act together, which would explain their synergistic effect on step 1. Cwc21p and Isy1p might be considered to function like alternative splicing factors, regulating the stringency of intron recognition under certain circumstances.

### Cwc21p as an alternative splicing factor

We propose a novel mechanism for alternative splicing and for the regulation of gene expression by preventing or promoting use of certain splice sites in response to environmental or metabolic changes. Consistent with Cwc21p being required under stress situations, the *CWC21* promoter contains a target sequence for Crz1p, a transcription factor activated by osmotic and temperature shock (54,55).

Some parallels may be drawn between the proposed mode of action of Cwc21p and SR (containing a serine and arginine rich ‘RS’ domain) protein function. SR proteins stabilize interactions in human spliceosomes and promote the use of weak splice sites. It has been proposed that *S. cerevisiae* does not require SR proteins as the 5′SS and BP sequences are highly conserved and interact more stably with the U1 and U2 snRNAs during spliceosome assembly (56). In support of this, certain mutations at the 5′SS or BP of a yeast pre-mRNA that reduce complementarity with the snRNAs could be compensated by tethering an RS-domain polypeptide nearby, whereas the RS-domain protein had no effect with a WT yeast pre-mRNA (57).

We propose that Cwc21p may similarly stabilize certain suboptimal pre-mRNA/U5 snRNA interactions at the catalytic centre of the spliceosome, especially under stressful metabolic or environmental conditions, such as heat stress. This suggests a mechanism for alternative splicing control that functions within the active spliceosome rather than during spliceosome assembly (6,58) and we propose that SRm300/SRRM2 may function in the same manner in the regulation of splicing in humans. This is compatible with SRm300 being the only SR protein found in the stable catalytic core of human spliceosome C complex, likely functioning after the first chemical reaction (2,28).

## SUPPLEMENTARY DATA

Supplementary Data are available at NAR Online.

SUPPLEMENTARY DATA
