# Supplementary material for: Cwc21p promotes the second step conformation of the spliceosome and modulates 3′ splice site selection
Source: Nucleic Acids Res. 2015 Mar 3;43(6):3309–17. doi: 10.1093/nar/gkv159 (PMC4381068; doi:10.1093/nar/gkv159)
Supplement: SUPPLEMENTARY DATA [file supp_43_6_3309__index.html]

Cwc21p promotes the second step conformation of the spliceosome and modulates 3′ splice site selection — Cwc21p promotes the second step conformation of the spliceosome and modulates 3′ splice site selection — SUPPLEMENTARY DATA 

# Cwc21p promotes the second step conformation of the spliceosome and modulates 3′ splice site selection

## SUPPLEMENTARY DATA

**Files in this Data Supplement:**

- SUPPLEMENTARY DATA
- SUPPLEMENTARY DATA
