# Supplementary material for: Cwc21p promotes the second step conformation of the spliceosome and modulates 3′ splice site selection
Source: Nucleic Acids Res. 2015 Mar 3;43(6):3309–17. doi: 10.1093/nar/gkv159 (PMC4381068; doi:10.1093/nar/gkv159)
Supplement: SUPPLEMENTARY DATA [file supp_gkv159_nar-00092-a-2015-File003.pdf]

SUPPLEMENTARY INFORMATION

Cwc21p promotes the second step conformation of the spliceosome and modulates 3' splice site selection.

Amit Gautam[1,4,#], Richard J. Grainger[1,#], J. Vilardell[2,3], J. David Barrass[1] and Jean D. Beggs[1,*]

[1]Wellcome Trust Centre for Cell Biology, University of Edinburgh, King's Buildings, Mayfield Road, Edinburgh, EH9 3BF, UK.

[2]Department of Molecular Genomics, Institute of Molecular Biology of Barcelona (IBMB), Barcelona, Spain.

[3]Institució Catalana de Recerca i Estudis Avançats (ICREA), Spain.

[4]Present address: Department of Medicine, Imperial College London, London W12 0NN, UK.

[#]equal contribution

*To whom correspondence should be addressed
Tel: +44-131-650-5351; Fax: +44-131-650-5351; e-mail: jbeggs@ed.ac.uk

CONTENTS

SUPPLEMENTARY TABLE S1. List of yeast strains and plasmids.

SUPPLEMENTARY FIGURE S1. Yeast two-hybrid interaction of Snu114p and Cwc21p.

SUPPLEMENTARY FIGURE S2. Examples of unusual splicing events in *cwc21Δ*.

SUPPLEMENTARY REFERENCES

Supplementary Table S1. List of yeast strains and plasmids.

Strain	Genotype	Source/reference
BY4741 (WT)	*MAT**a**, his3Δ1; leu2Δ0; met15Δ0; ura3Δ0*	Euroscarf
Y04316-cwc21Δ	*MAT**a**, his3Δ1 leu2Δ0 lys2Δ0 ura3Δ0, cwc21Δ::KanMX4*	Euroscarf
L40ΔG	*MAT**a** his3Δ200 trp1-901 leu2-3,112 ade2 LYS2::(4lexAop-HIS3) URA3::(8lexAop-lacZ) Δgal4::KAN*	(1)
yJU75-8+	*MAT**a**, ade2 cup1Δ::ura3 his3 leu2 lys2 prp8Δ::LYS2 trp1, pMK8-1 (PRP8-wt TRP CEN ARS)*	(2)
yJU75-8-WT-21Δ	*MAT**a**, ade2 cup1Δ::ura3 his3 leu2 lys2 prp8Δ::LYS2 trp1, pMK8-1 (PRP8-wt TRP CEN ARS), cwc21Δ::HPH-NT1*	This work
U5KO	*MAT**a**; ura3-52; trp1Δ63; leu2Δ1; his3Δ200; GAL2; snr7Δ::kanMX6; pRS416-U5*	(3)
U5KO-21Δ	*MAT**a**; ura3-52; trp1Δ63; leu2Δ1; his3Δ200; GAL2; snr7Δ::kanMX6; pRS416-U5, cwc21Δ::HPH-NT1*	This work
PRP16KO	*Mat**a**, prp16Δ:Hph-NT1, leu2-3,112 trp1-1 can1-100 ura3-1 ade2-1 his3-11,15*, pRS416-P_{met25}-PRP16,	(4)
PRP16KO -21Δ	*Mat**a**, prp16Δ:Hph-NT1, leu2-3,112 trp1-1 can1-100 ura3-1 ade2-1 his3-11,15*, pRS416-P_{met25}-PRP16, *cwc21Δ::NAT-NT2*	This work
yTB23	*MATa, lys2Δ, his3Δ, leu2Δ, ura3Δ, met15, snu114::KanMX, pTB1*	(5)
yRG23	*MATa, lys2Δ, his3Δ, leu2Δ, ura3Δ, met15, snu114::KanMX, pTB1, cwc21::HPH-NT1*	This work
**Plasmid**	**Description**	**Source/reference**
pFA6a-NatNT2	Nourseothricin knockout vector	PCR toolbox, Euroscarf
pFA6a-HphNT1	Hygromycin knockout vector	PCR toolbox, Euroscarf
p*PRP8* series	pRS314-*PRP8-WT, TRP, CEN, ARS*	(2)
	pRS314-*prp8-R1753K, TRP, CEN, ARS*	(2)
	pRS314-*prp8-162, TRP, CEN, ARS*	(2)
	pRS314-*prp8-syf77, TRP, CEN, ARS*	(2)
	pRS314-*prp8-161, TRP, CEN, ARS*	(2)
	pRS314-*prp8-156, TRP, CEN, ARS*	(2)
p*SNU114* series	pRS315-*SNU114, LEU2, ARS, CEN*	(5)
	pRS315-*snu114- 40, LEU2, ARS, CEN*	(5)
pBTM116	2μ, *TRP1*, lexA(1-202)-BD	(6)
pBTM116-Snu114	2μ, *TRP1*, lexA(1-202)-BD-Snu114 (Full length)	This work
pBTM116-Snu114 –IVa+IVb+V	2μ, *TRP1*, lexA(1-202)-BD-Snu114 (domains IVa (aa692-aa860) +IVb+V	This work
pBTM116-Snu114 –IVa+V	2μ, *TRP1*, lexA(1-202)-BD-Snu114 (domains IVa (aa692-aa860) +V	This work
pBTM116-Snu114 –IVb+V	2μ, *TRP1*, lexA(1-202)-BD-Snu114 (domains (IVb+V)	This work
pBTM116-Snu114 –IVa	2μ, *TRP1*, lexA(1-202)-BD-Snu114 domains IVa	This work
pBTM116-Snu114 –IVb	2μ, *TRP1*, lexA(1-202)-BD-Snu114 domains IVb	This work
pBTM116-Snu114 –V	2μ, *TRP1*, lexA(1-202)-BD-Snu114 domains V	This work
pACTII	2μ, LEU2, Gal4-AD	(7)
pACTII-Cwc21	2μ, *LEU2*, Gal4-AD-*CWC21* (full length)	(8)
pU5 snRNA-series	pRS416-*U5-WT, URA3, CEN, ARS*	(3)
	pRS314-*U5-1, TRP1, CEN, ARS*	(3)
	pRS314-*U5-2, TRP1, CEN, ARS*	(3)
	pRS314-*U5-3, TRP1, CEN, ARS*	(3)
	pRS314-*U5-Δ94/95, TRP1, CEN, ARS*	(3)
	pRS314-*U5-Ins 1U 94/95, TRP1, CEN, ARS*	(3)
	pRS314-*U5-Δ96/97, TRP1, CEN, ARS*	(3)
	pRS314-*U5-ΔG93, TRP1, CEN, ARS*	(3)
	pRS314-*U5-Ins 1U 93/94, TRP1, CEN, ARS*	(3)
pPrp16 series	pRS416-P_{met25}-*PRP16, URA3, CEN, ARS*	(4)
	pRS314-*PRP16, TRP1, CEN, ARS*	(4)
	pRS314-*prp16-R686I, TRP1, CEN, ARS*	(4)

	pRS314-*prp16-L335F*, *TRP1, CEN, ARS*	This work
	pRS314-*prp16-K379R*, *TRP1, CEN, ARS*	This work
	pRS314-*prp16-Q685H*, *TRP1, CEN, ARS*	(4)
	pRS314-*prp16-302*, *TRP1, CEN, ARS*	(4)
	pRS314-*prp16-R686Q*, *TRP1, CEN, ARS*	This work
	pRS314-*prp16-Y386D*, *TRP1, CEN, ARS*	This work
	pRS314-*prp16-C862Y*, *TRP1, CEN, ARS*	This work
	pRS314-*prp16-201*, *TRP1, CEN, ARS*	(4)
	pRS314-*prp16-202*, *TRP1, CEN, ARS*	(4)
Copper reporters	pMA-WT	(2)
	pMA-A3C	(2)
	pMA-BS-G	(2)
	pMA-BS-C	(2)
	pMM-WT	(2)
	pMM-3'UuG	(2)

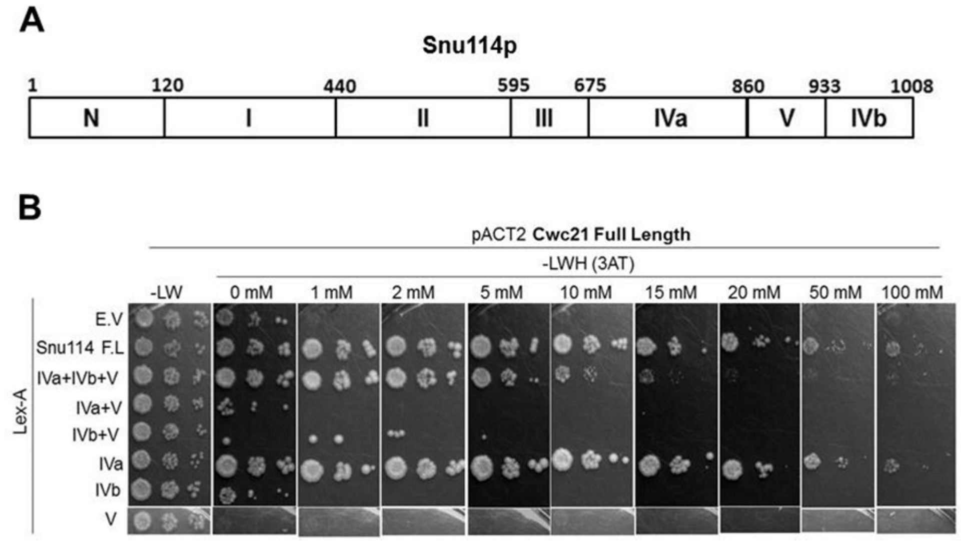

Supplementary Figure S1. Yeast two-hybrid interaction of Snu114p and Cwc21p. **(A)** Domain structure of Snu114p. **(B)** Interaction of full-length Cwc21p as a pACT2 fusion (pACT2-Cwc21) with different Snu114p LexA-DNA binding domain constructs on drop-out (lacking leucine (L), tryprophan (W) and/or histidine (H)) plates with different concentrations of 3-aminotriazole (3-AT). The cells were diluted to OD = 0.3 and two tenfold serial dilutions, spotted and grown at 30ºC for at least 2 days. F.L is full length; roman numeral indicate the domains of Snu114p present, as in Figure 1A.

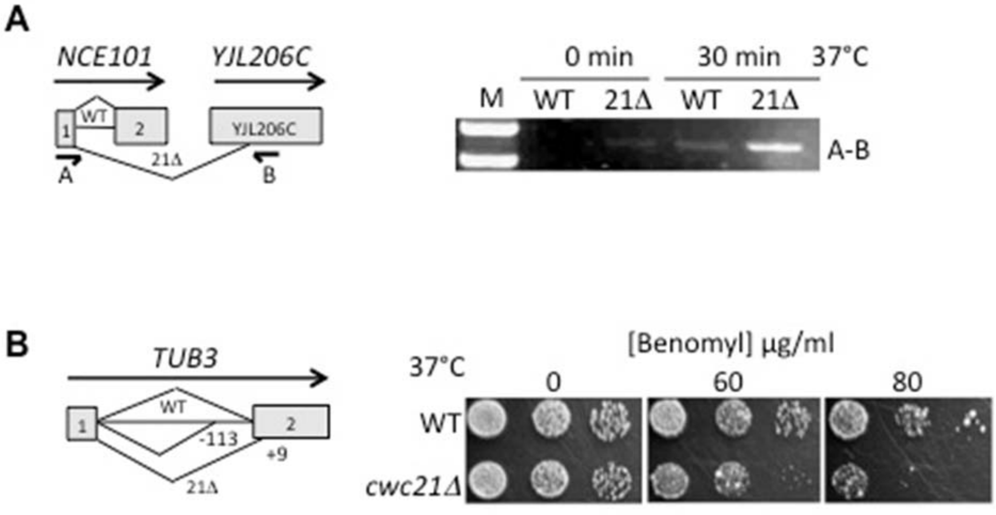

Supplementary Figure S2. Examples of unusual splicing events in *cwc21Δ*.

(A) RT-PCR validation of splicing of *NCE101* exon 1 to a non-canonical 3'SS in YJL206C. Left: schematic of the genes as in Fig. 7. Arrows A and B indicate the positions of PCR primers. Right: agarose gel electrophoresis of RT-PCR products from WT and *cwc21Δ* (*21Δ*) cells grown at 30°C (0 min) or shifted to 37°C for 30 min.

(B) Left: schematic of *TUB3* alternative splicing, showing the 3'SS detected by microarray analysis (lower; 9 bases downstream of the canonical 3'SS) and an additional 3'SS identified by cDNA sequencing (113 bases upstream of the canonical 3'SS), Right: consistent with a defect in splicing *TUB3* transcripts, sensitivity to the microtubule toxin benomyl is increased in *cwc21Δ* relative to WT at 37°C.

SUPPLEMENTARY REFERENCES

1. Fromont-Racine,M., Mayes,A.E., Brunet-Simon,A., Rain,J.C., Colley,A., Dix,I., Decourty,L., Joly,N., Ricard,F., Beggs,J.D., *et al.* (2000) Genome-wide protein interaction screens reveal functional networks involving Sm-like proteins. *Yeast Chichester Engl.*, **17**, 95–110.

2. Liu,L., Query,C.C. and Konarska,M.M. (2007) Opposing classes of prp8 alleles modulate the transition between the catalytic steps of pre-mRNA splicing. *Nat. Struct. Mol. Biol.*, **14**, 519–526.

3. Kershaw,C.J., Barrass,J.D., Beggs,J.D. and O'Keefe,R.T. (2009) Mutations in the U5 snRNA result in altered splicing of subsets of pre-mRNAs and reduced stability of Prp8. *RNA*, **15**, 1292–1304.

4. Hahn,D., Kudla,G., Tollervey,D. and Beggs,J.D. (2012) Brr2p-mediated conformational rearrangements in the spliceosome during activation and substrate repositioning. *Genes Dev.*, **26**, 2408–2421.

5. Brenner,T.J. and Guthrie,C. (2005) Genetic analysis reveals a role for the C terminus of the Saccharomyces cerevisiae GTPase Snu114 during spliceosome activation. *Genetics*, **170**, 1063–1080.

6. Bartel,P., Chien,C.T., Sternglanz,R. and Fields,S. (1993) Elimination of false positives that arise in using the two-hybrid system. *BioTechniques*, **14**, 920–924.

7. Fromont-Racine,M., Rain,J.C. and Legrain,P. (1997) Toward a functional analysis of the yeast genome through exhaustive two-hybrid screens. *Nat. Genet.*, **16**, 277–282.

8. Grainger,R.J., Barrass,J.D., Jacquier,A., Rain,J.-C. and Beggs,J.D. (2009) Physical and genetic interactions of yeast Cwc21p, an ortholog of human SRm300/SRRM2, suggest a role at the catalytic center of the spliceosome. *RNA*, **15**, 2161–2173.
